# Implementation of the Evidence-Based Practice in Psychology in Ukraine: Prediction Model Development

**DOI:** 10.5964/ejop.14559

**Published:** 2026-02-27

**Authors:** Mariana Velykodna, Gelena Lazos, Liudmyla Karamushka, Ivan Klymenko, Vladyslav Deputatov, Tetiana Pysarenko

**Affiliations:** 1 Psychology Department, Ukraine Sigmund Freud University, Kyiv, Ukraine; 2 Practical Psychology Department, Kryvyi Rih State Pedagogical University, Kryvyi Rih, Ukraine; 3 Laboratory of Organizational Psychology, G.S. Kostiuk Institute of Psychology of the National Academy of Educational Sciences of Ukraine, Kyiv, Ukraine; 4 Psychology Department, State University of Economics and Technology, Kryvyi Rih, Ukraine; Università Cattolica del Sacro Cuore; Milan, Italy

**Keywords:** attitudes, evidence-based practice, evidence-based practice in psychology, knowledge, mental health, psychology

## Abstract

**Objective**: By 2021, researchers reported limited access to evidence-based mental health interventions in Ukraine, which became more crucial after the 2022 Russian invasion of Ukraine. Evidence-based practice in psychology (EBPP) requires professionals to consider equal contributions of the best available research, practical experience, and the context of the unique client. This study aimed to reveal the possible predictors of EBPP implementation among Ukrainian psychologists (*n* = 366) by developing and testing multivariable prediction models. **Methods**: The research design followed the methodology of “Transparent reporting of a multivariable prediction model for individual prognosis or diagnosis” — TRIPOD checklist. The online survey included a questionnaire on sociodemographic characteristics, educational and professional background, and a purposely developed part regarding the knowledge, attitudes, and utilization of the EBPP approach. **Results**: Regression analysis revealed different prediction models for the belief that the respondent implements EBPP and the intensity of EBPP elements implementation, explaining 24.4% and 18.5% of their variance with 3 and 5 predictors, respectively. **Conclusion**: Psychologists believed they were implementing EBPP when they used interventions/methods with proven efficacy, were members of psychological associations, and assessed their knowledge regarding EBPP higher. However, more intensive use of EBPP elements was predicted by a psychologist’s experience in personal therapy and supervision, a positive attitude toward EBPP elements, membership in a psychological association, and a perceived level of knowledge regarding EBPP. The belief that the psychologist’s practice aligns with the EBPP requirements and the intensity of using EBPP elements had a rather moderate correlation.

Evidence-based practice in psychology (EBPP) was designed to enforce outcomes of psychological interventions by integrating the best available research, clinical experience extracted from practice, and a client’s specifics, including culture and values (APA, 2006) as equally important components in the professional’s decision-making. The EBPP promotion, first initiated by the American Psychological Association and supported by the Canadian Psychological Association, valuably contributed to the ongoing worldwide discussion of:

1. What the best research is and what the psychologist’s ability to understand it critically is required (APA, 2006; CPA, 2012; Middleton et al., 2020).
2. How the evidence could be implemented in practice or learned from practice (Boswell & Schwartzman, 2024; Waltman et al., 2020).
3. What specifics of clients should be addressed by tailoring psychological interventions and how (Melgarejo et al., 2020; Pfeiffer et al., 2023).

Among the advantages of the EBPP, practitioners mention its impact in promoting mental health and effective psychological practice, and in outcome improvement (Middleton et al., 2020) as well as clarifying psychologists’ roles in collecting, summarizing, and implementing the research evidence in regard to practice (Spring & Neville, 2010). Among the disadvantages and barriers to EBPP implementation, scholars notice its possible excessive scientization, which means making it too complicated for practical understanding because of using scientific terms (Berg, 2019), and less valued practice-based evidence (i.e., evidence extracted from practice) compared to research findings (Boswell & Schwartzman, 2024) prevalent or set by policies in specific countries. Practitioners also reported that implementing the EBPP approach may be challenging due to the lack of time and money for updating knowledge (Meyer et al., 2020) and the non-applicability of research extracted from ideal conditions of randomized controlled trials (RCT) to real clinical practice (Nelson et al., 2006).

Research reveals that a lack of knowledge of what exactly the EBPP is leads to psychologists’ resistance to its implementation (Klymenko et al., 2025; Lilienfeld et al., 2013). No less important is that previous research demonstrated that the lack of sufficient knowledge regarding the EBPP could be manifested in confusing it with evidence-based treatments (EBT), i.e., specific interventions or methods with proven efficacy that are not necessarily practiced by a specific mental health professional using the frame of the EBPP (DiMeo et al., 2012; Newburg, 2020), which was also observed in Ukraine (Lazos et al., 2024). Although utilizing EBT or so-called empirically supported treatments (ESTs) plays a valuable role in the practice’s relevance to research (Shedler, 2018), its confusion with EBPP may pose a risk of developing a psychologist’s belief that they implement EBPP while practicing any method with proven efficacy. Instead, within the EBPP approach, a psychologist should not overestimate or underestimate EBT compared to a client’s values and preferences or their own practical experience (Berg, 2020) but consider them as equal contributions to the process of decision-making (Newburg, 2020).

In Ukraine, research revealed a lack of knowledge regarding evidence-based practice before the 2022 Russian invasion (Hook et al., 2021) and after it (Velykodna et al., 2025). The few works published in Ukraine devoted to this topic have focused only on EBT (e.g., Chaban, 2019; Sochenko & Gabińska, 2017; Velykodna, 2023). The 2022 Russian invasion of Ukraine resulted in a mental health crisis associated with both the terrible consequences of war (Lushchak et al., 2024; Pavlova et al., 2024) and the unpreparedness of the mental health care system (Seleznova et al., 2023). Widespread traumatization among Ukrainian children and adults required appropriate evidence-based solutions (Pfeiffer et al., 2023). To address this gap, in 2022–2023, various psychological associations in Ukraine collaborated with EBPP experts from abroad to provide their members with lectures on EBPP (Palii et al., 2024).

In 2024, the Order of the Ministry of Health of Ukraine (January 25, 2024, No. 129/41474), for the first time, defined a list of psychotherapy methods (interventions, techniques) with proven efficacy relevant to a particular disorder or condition, as well as recommended tools for psychological assessment, legal to be implemented in Ukraine (Ministry of Health of Ukraine, 2024). However, this solution legitimates and promotes the EBT, while EBPP has no official status to date of research. As a result, the prevalence of EBPP implementation in Ukraine, as well as the facilitating factors and barriers, are not yet known.

Therefore, this paper focuses on revealing the possible predictors of EBPP implementation among Ukrainian psychologists by developing and testing multivariable prediction models regarding its cognitive and behavioral aspects, which would contribute to planning further dissemination of EBPP in Ukraine.

## Method

### Study Design

This study followed the TRIPOD Checklist for Prediction Model Development (Collins et al., 2015) in organizing and reporting procedures.

#### Theoretical Framework

**Figure 1:**
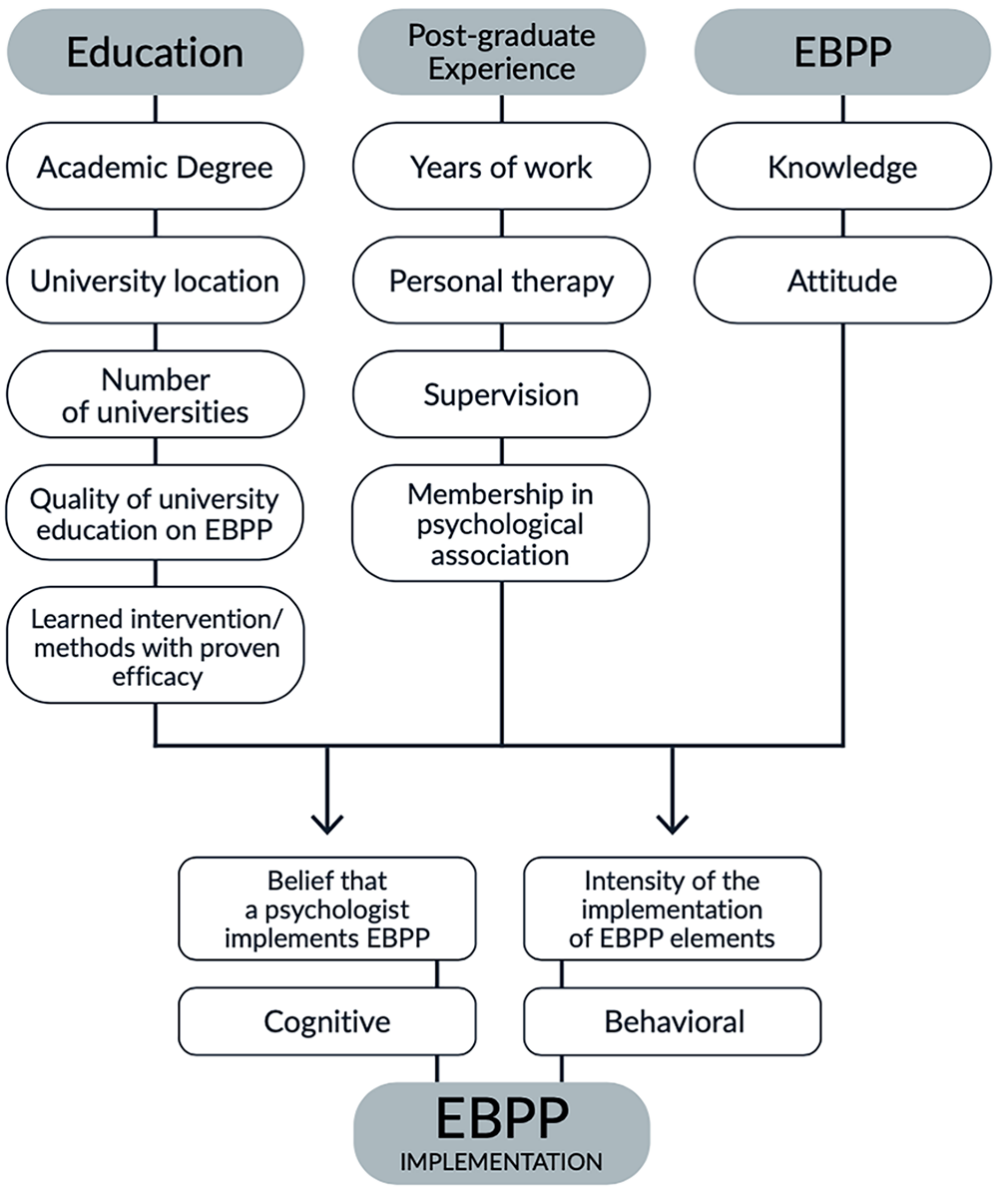
Theoretical Framework

We developed a theoretical framework indicating a psychologist’s *Belief that they implement EBPP* and the *Intensity of the implementation of EBPP* as outcomes in this study, representing cognitive and behavioral aspects of EBPP implementation. Perceived quality of university education regarding EBPP, number and location (i.e., in Ukraine or abroad) of graduated universities, attitudes to EBPP elements, perceived level of knowledge regarding EBPP, work experience, membership with a psychological association, experience to be a client and supervisee, being trained to use a method with proven efficacy, age, and gender were tested as potential predictors in this study (Figure 1). All the potential predictors were grouped as representing the Educational background, Post-graduate experience, and EBPP utilization experience extracted from the literature review.

#### Research Questions

Following the theoretical framework, we aimed to develop multivariable prediction models for the psychologists’ *Belief that they implement EBPP* and the *Intensity of the implementation of EBPP* with three research questions:

1. What prediction model better forecasts the *Belief of Ukrainian psychologists that they implement EBPP*?
2. What prediction model better prognoses the *Intensity of the implementation of EBPP* among Ukrainian psychologists?
3. What is the relationship between the *Belief that they implement EBPP* and the *Intensity of the implementation of EBPP* in this sample?

### Participants

Ukrainian psychologists who hold a university diploma in psychology and conduct psychological practice in its broad meaning (e.g., all types of professional activities of a psychologist allowed in Ukraine, such as clinical assessment, research, counseling, low-intensive and crisis interventions, psychoeducation, and, with appropriate additional education, psychotherapy) were invited to participate voluntarily in this study. We aimed to attract participants with different backgrounds and from different regions of Ukraine, which was monitored during the data collection process.

### Survey Development

Considering wartime, an online survey was considered appropriate for this study, as it allowed Ukrainian psychologists from different locations to be contacted in a short time frame and provided confidentiality. A questionnaire included sociodemographic data, educational background, various working experiences, and purposely developed questions to measure EBPP attitudes and implementation. Most of the questions regarding EBPP (e.g., quality of university education, perceived level of knowledge regarding EBPP, attitudes to EBPP elements as valuable or not, and intensity of their implementation) were formulated as 7-point Likert scales. All variables and relevant data types are listed in Table A1 of the Appendix.

#### Belief That a Psychologist Implements EBPP

This variable was measured using a 5-point Likert scale to the question, “To what extent do you implement the EBPP approach?” (1 – Not at all; 5 – To a large extent). Additionally, this question was provided with a response option “I do not know” (0), which allowed identifying respondents who did not know what EBPP was and excluding them from the development of the prediction model of this outcome.

#### Intensity of the Implementation of EBPP

To assess the *Intensity of the implementation of EBPP* elements, we purposely developed a 6-item scale with each two items representing one theorizing EBPP element and tested its factor structure (See Table 1). The respondents assessed the items with a 7-point Likert scale regarding how often they implement the listed EBPP elements as follows: Daily (7); Several times a week (6); Several times a month (5); Several times a year (4); Annually (3); Once every two to five years (2); Less than once in five years (1). As an outcome of this study, we used a coefficient calculated as the sum of these scales divided by the number of items (6). Therefore, the index of *Intensity of the implementation of EBPP* could be scored from 1 to 7 for each survey participant.

**Table 1:** Confirmatory Factor Analysis (CFA) of the Developed Scale for the Intensity of the Implementation of EBPP Elements Assessment

		CFA				
Scale	Items	*CFI*	*TLI*	*RMSEA*	*CI 95%*	*p-value*
Considering the best available research.	Updating knowledge about the possibilities and limits of using a particular method or intervention.	.996	.991	.035	.00–.087	<.001
	Updating knowledge about the peculiarities of working with certain issues, requests, and personality types.					<.001
Considering practical experience.	Analysis of own practical experience (e.g., through supervision and discussions with colleagues).					<.001
	Analysis of the practical experience of colleagues (e.g., through supervision, discussions with colleagues, and clinical ateliers).					<.001
Considering the client's sociocultural specifics and preferences.	Taking into account the sociocultural characteristics of a particular client (and appropriate choice or modification of the approach, method, and technique).					<.001
	Taking into account the personal preferences of the client (and the appropriate choice or modification of the approach, method, and technique).					<.001

### Data Collection

The invitation for participation in the survey was distributed online through emails and social media platforms of the largest psychologists’ associations in Ukraine (National Psychological Association, Ukrainian Union of Psychotherapists). Psychologists were invited to participate voluntarily in the survey regarding their attitudes to EBPP and could withdraw from participation at any time. The survey responses were collected from December 14, 2023, till March 7, 2024, via the SurveyMonkey platform. Written consent forms for participation were collected along with survey responses.

Overall, 449 Ukrainian psychologists confirmed they received the invitation to fill in the survey. Among them, 81.5% (*n* = 366) provided complete responses used in data analysis. Incomplete responses were excluded from data analysis.

### Data Analysis

Data analysis included descriptive statistics, confirmatory factor analysis (CFA), linear regression analysis, and Pearson correlation calculation using the R-based software Jamovi 2.3.28. The data and Jamovi logs are available at the Open Science Framework repository at Velykodna and Deputatov (2024).

## Results

### Participants

Overall, 366 Ukrainian psychologists aged 21 to 67 (*M* = 38.3; *SD* = 9.54) with 1–37 years of work experience (*M* = 8.4; *SD* = 7.03) provided complete responses to the questionnaire. Most of them were middle-career females currently located in Ukraine who had graduated from Ukrainian universities. The full sociodemographic characteristics gathered from the survey are presented in Table 2.

**Table 2:** Sociodemographic Characteristics and Professional Background of the Sample

Characteristic	*n*	*%*
Gender		
Male	28	7.7
Female	338	92.3
Current residence		
In Ukraine	301	82.2
Abroad	65	17.8
Academic degrees in Psychology (multiple choice)		
Bachelor	105	28.69
Master	262	71.58
Doctoral Degree	44	12.02
Field of practice (multiple choice)		
Education	77	21.04
Science	22	6.01
Mental Health	48	13.11
Social work	69	18.85
Military	18	4.92
Law	8	2.19
Business	21	5.74
Private practice	249	68.03
Other	2	0.55
Learned method(s) with proven efficacy		
Yes	282	89.2
No	34	10.8
Membership in any psychological association		
Yes	249	68
No	117	32
Personal therapy experience		
Yes	294	93
No	22	7
Less than 50 hours	43	13.61
51 to 100 hours	56	17.72
101 to 200 hours	61	19.3
201 to 300 hours	50	15.82
More than 300 hours	84	26.58
Supervision experience		
Yes	292	92.4
No	24	7.6
Less than 50 hours	93	29.43
50 to 100 hours	64	20.25
100 to 200 hours	63	19.94
200 to 300 hours	28	8.86
More than 300 hours	44	13.92
Location of graduated universities (multiple choice)		
In Ukraine	357	97.5
Abroad	29	7.9
	*M (SD)*	*Me (min-max)*
Age	38.3 (9.54)	38 (21–67)
Number of graduated universities	1.54 (.383)	1 (1–5)
Work experience (in years)	8.4 (7.03)	5 (1–37)

Most respondents held master’s diplomas in psychology (71.58%), were members of psychological societies (68.03%), and were trained to provide at least one intervention/psychotherapy method with proven efficacy (77.05%). Among the 29 methods and interventions, psychologists mentioned psychoanalytic psychotherapy (23.50%), art therapy (21.31%), cognitive-behavioral (21.31%), and gestalt therapy (17.76%) most often.

The variance of predictors and outcomes within the sample is presented in Table 3.

**Table 3:** Descriptive Statistics of all Variables (Measured Predictors and Outcomes)

Variables	*Range (Min–Max)*	*M*	*SD*	*SE*	*Me*
Quality of university education regarding EBPP.	1–7	4	1.71	.09	4
Perceived level of knowledge regarding EBPP.	1–7	5.11	1.31	.07	5
Attitude toward EBPP elements					
Updating knowledge about the possibilities and limits of using a particular method or intervention.	1–7	6.44	.85	.04	7
Updating knowledge about the peculiarities of working with certain issues, requests, and personality types.	1–7	6.55	.73	.04	7
Analysis of own practical experience (e.g., through supervision and discussions with colleagues).	1–7	6.65	.74	.04	7
Analysis of the practical experience of colleagues (e.g., through supervision, discussions with colleagues, and clinical ateliers).	2–7	6.33	.85	.04	7
Taking into account the sociocultural characteristics of a particular client (and appropriate choice or modification of the approach, method, and technique).	1–7	6.19	.98	.05	6
Taking into account the personal preferences of the client (and the appropriate choice or modification of the approach, method, and technique).	1–7	5.86	1.15	.06	6
Considering the best available research scale.	3–14	12.99	1.44	.08	14
Considering the practical experience scale.	3–14	12.98	1.46	.08	14
Considering the client's sociocultural specifics and preferences scale.	2–14	12.05	1.92	.1	12
Index of Attitude toward EBPP elements (Overall/6).	2.33–7	6.34	.65	.03	6.5
Belief that a psychologist implements EBPP.	1–5	3.92	0.99	0.05	4
	0 (‘I do not know’)	*n* = 26	7.1%		
Intensity of the implementation of EBPP elements					
Updating knowledge about the possibilities and limits of using a particular method or intervention.	1–7	4.26	1.16	.06	4
Updating knowledge about the peculiarities of working with certain issues, requests, and personality types.	2–7	4.42	1.03	.05	4
Analysis of own practical experience (e.g., through supervision and discussions with colleagues).	1–7	4.59	1.02	.05	5
Analysis of the practical experience of colleagues (e.g., through supervision, discussions with colleagues, and clinical ateliers).	1–7	4.54	1	.05	5
Taking into account the sociocultural characteristics of a particular client (and appropriate choice or modification of the approach, method, and technique).	1–7	4.58	1.44	.08	5
Taking into account the personal preferences of the client (and the appropriate choice or modification of the approach, method, and technique).	1–7	4.97	1.42	.07	5
Considering the best available research scale.	3–14	8.69	1.97	.1	8
Considering the practical experience scale.	2–14	9.14	1.87	.1	10
Considering the client's sociocultural specifics and preferences scale.	2–14	9.54	2.62	.14	10
Index of the intensity of the implementation of EBPP elements (Overall/6).	1.83–6.83	4.56	0.78	.04	4.67

Interestingly, 1.64% of the surveyed psychologists assessed their knowledge regarding EBPP as ‘nothing’ and 4.1% as ‘almost nothing’, while 7.1% responded that they did not know if they implemented EBPP. Pearson correlation analysis revealed a moderate relationship between the *Belief that a psychologist* implements EBPP and the *Intensity of EBPP elements implementation by them* (*r* = .196, *p* < .001; *CI* 95%: .082–.304).

### Models Development

The development of prediction models was performed as follows. The hypothesized models proposed in the theoretical framework (Figure 1) were tested via linear regression analysis by adding variables one by one as possible predictors to the calculation. Each tested variable remained in the prediction model or was rejected depending on its p-value (*p* < .05 was estimated as a cutoff for this process). Changes in the *p*-value of other model variables were also considered when adding each subsequent predictor. If adding a new statistically significant predictor made the previously added predictors insignificant and vice versa, the predictor with a larger β-value that resulted in a larger *R*^2^ remained in the model.

### Models’ Performance

Following the described procedure, we developed two prediction models for the *Belief that a psychologist implements EBPP* and the *Intensity of EBPP elements implementation*, explaining 24.4% (*F* = 30.8, *p* < .001, adjusted *R*^2^ = .236) and 18.5% (*F* = 14.1, *p* < .001, adjusted *R*^2^ = .172) of their variance with 3 and 5 predictors, respectively. Two variables — *Membership in psychological association* and the *Perceived level of knowledge regarding EBPP* — significantly contributed to both models.

The developed prediction models for the *Belief that a psychologist implements EBPP* and the *Intensity of EBPP elements implementation* were summarized in Table 4.

**Table 4:** Regression Coefficients

			95% CI		
Variable	Estimate (β)	*SE*	[LL, UL]	*p*
Model 1. Belief that a psychologist implements EBPP				
Intercept.	1.437	.29	.866, 2.008	<.001
Learned method with proven efficacy.	.908	0.194	.526, 1.29	<.001
Membership in a psychological association.	.501	.127	.25, .751	<.001
Perceived knowledge regarding EBPP.	.237	.046	.146, .327	<.001
Model 2. Intensity of implementing EBPP					
Intercept.	1.516	.444	.643, 2.389	<.001
Supervision experience.	.367	.167	.038, .696	.029
Positive attitude to EBPP.	.254	.065	.127, .382	<.001
Membership in a psychological association.	.236	.101	.038, .435	.02
Perceived knowledge regarding EBPP.	.103	.034	.036, .17	.003
Personal therapy experience.	.41	.167	.081, .739	.015

*Note*. *CI* = Confidence Interval; LL = Lower Limit; UL = Upper Limit.

The strongest variable that predicted psychologists’ *Belief that they implement EBPP* was that they provided *Interventions/methods with proven efficacy* (β = .908, *p* < .001). Almost two times less influential was the psychologist’s *Membership in psychological association* (β = .501, *p* < .001), followed by the *Perceived level of knowledge regarding EBPP* (β = .237, *p* < .001). Instead, the strongest predictors for the *Intensity of EBPP elements implementation* were *Personal therapy of the psychologist* (β = .41, *p* = .015) and *Supervision* (β = .367, *p* = .029), which, however, had rather large standard error and *p*-value. They were followed by a *Positive attitude to EBPP elements* (β = .254, *p* < .001) and *Membership in psychological association* (β = .236, *p* = .02). The *Perceived level of knowledge regarding EBPP* (β = .101, *p* = .003) was performed as the weakest but significant predictor in this model.

Interestingly, work experience and educational background (e.g., number and location of universities, satisfaction with the quality of university education regarding EBPP), as well as age and gender, did not predict the studied outcomes in this sample.

## Discussion

Current challenges in providing mental health care in Ukraine after the 2022 Russian invasion encompass accessibility, continuity, and integrity issues (Palii et al., 2024). EBPP utilization is considered empowering psychologists in their clinical thinking and decision-making aimed at offering the best available intervention adjusted to a specific client (Lazos et al., 2024).

This study aimed to reveal the predictors of cognitive and behavioral aspects of EBPP implementation among Ukrainian psychologists using the theoretical framework extracted from a literature review. Within the theoretical framework, we tested a list of variables grouped as relevant to Educational background, Post-graduate experience, and EBPP Knowledge and Attitudes in predicting EBPP practice. The obtained results succeeded in predicting 24.4% of the variance of psychologists’ *Beliefs that they implement EBPP* and 18.5% of the *Intensity of EBPP elements implementation,* which is considered acceptable for social science research (Ozili, 2023). However, further research is needed, focusing on seeking possible additional predictors of the EBPP implementation among Ukrainian psychologists extracted from qualitative data, which would be more appropriate considering the result that about 7% of the surveyed psychologists knew very little about EBPP and were not sure if they implemented it or not to any extent. No less important is that the current study's findings contribute to the evidence that cognitive and behavioral aspects of EBPP implementation do not necessarily match (e.g., Jiménez-Pérez & Vargas-Contreras, 2020) and reveal some specifics of their predictors as follows.

Generally, the final developed prediction models included 6 variables related to the respondents’ *Education* (being trained to utilize EBT), *Post-graduate Experience* (personal therapy, supervision, membership in psychological association), as well as Attitudes and Knowledge regarding the *EBPP,* discussed below in order from the most to the least influential.

### Utilization of EBT (i.e., Interventions/Methods With Proven Efficacy)

Utilization of *Interventions/methods with proven efficacy* was the strongest predictor of psychologists’ *Belief that they implement EBPP*, while not predicting the *Intensity of EBPP elements implementation* in practice. First, this finding may reflect the well-known confusion of EBPP and EBT revealed in previous studies (DiMeo et al., 2012; Newburg, 2020; Wilson et al., 2009). Second, we suppose that it may highlight that the EBT training programs may be seen as potentially contradictory to the EBPP approach generally, at least in Ukrainian educational curricula (e.g., by developing a psychologist’s impression that their interventions are already *evidence-based enough* as they utilize EBT). However, this point requires further research among Ukrainian psychologists.

### Personal Therapy and Supervision

Unlike psychologists’ *Belief that they implement EBPP,* the strongest predictors for the *Intensity of EBPP elements implementation* were the *Personal therapy* of the psychologist and receiving *Supervision*. Although previous research suggests that an experience of personal therapy does not predict the effectiveness of psychologists’ practice (Moe & Thimm, 2021), it is seen as fundamental for professional training, identity development, and self-care of professionals (Norcross & VandenBos, 2018), including for learning EBTs (Orlinsky et al., 2005). Similarly, there is growing evidence of the contribution of supervision to EBPP training outcomes (Barrett et al., 2020; Borders et al., 2023). Therefore, we assume that *Personal therapy* and *Supervision* as a part of psychologists’ training may be seen as valuable contributors to further EBPP implementation in practice.

### Positive Attitudes to EBPP Elements

*Positive attitudes to EBPP elements* also predicted the *Intensity of EBPP elements implementation* in our study. This finding aligns with previous research focused on the attitudes and implementation of EBPP. For instance, a recent review of 46 studies empirically examining factors associated with implementing science into practice revealed that most papers reported a strong influence of attitudes on it (Fishman et al., 2021). Data collected among Australian psychologists also showed that positive attitudes to EBPP influenced practice but were also moderated by gender and shorter work experience (Hamill & Wiener, 2018). In Portugal, research revealed that psychologists were more likely to use specific interventions when had a positive attitude toward them (Mendes-Santos et al., 2020). However, this result differs from previous findings from German-speaking countries where positive attitudes toward EBPP did not correspond to the higher implementation of its elements, e.g., reviewing evidence, associated with a lack of time (Frytz et al., 2022). Further research would benefit from investigating the specification of attitudes to EBPP elements regarding the appeal, requirements, openness, and divergence, as proposed by Aarons and colleagues (2010).

### Membership in Psychological Associations

In our study, *Membership in Psychological Associations* significantly contributed to both outcomes — psychologists’ *Belief that they implement EBPP* and the *Intensity of EBPP elements implementation*. First, this finding could be seen in the light of the history of EBPP, which shows that professional psychological associations play a significant role in its dissemination and implementation in a specific country, including by officially formulated requirements for psychologists’ practice (e.g., APA, 2006; CPA, 2012). Second, research revealed that psychologists consider professional standards and publications on EBPP provided by professional institutions as supporters for EBPP implementation (Flegge, 2023). No less important is that In Ukraine, psychological associations currently contribute to EBPP promotion by offering EBT training and occasional brief advanced training regarding EBPP. It is important because considering the best available research, which is a part of EBPP, requires lifelong education of psychologists (Spring, 2007). This finding also highlights the potential benefits of engaging professional associations in Ukraine in EBPP promotion.

### Perceived Level of Knowledge Regarding EBPP

The *Perceived level of knowledge regarding EBPP* slightly but significantly affected both outcomes assessed in this study. First, this finding may be associated with the specificity of this variable as it was based on psychologists’ self-assessment of their knowledge, and it should be recalled that, as previous research revealed, psychologists tend to assess their knowledge of EBPP higher than their colleagues’ (Middleton et al., 2020). However, even those assessed as skilled in scientific data analysis by independent observers were not necessarily able to use their knowledge during the very interventions (Jiménez-Pérez & Vargas-Contreras, 2020). Nevertheless, previous research suggests that education regarding EBPP results in increased knowledge and improved attitudes toward EBPP (Lim et al., 2012), while more intensive training models lead to the implementation of EBPP in a psychologist’s behavior (Frank et al., 2020). Future research should be focused on what exactly is known regarding EBPP among Ukrainian psychologists.

### Other Findings

Interestingly, the *Perceived quality of university education regarding EBPP* did not predict the implementation of EBPP, while previous research reported that learning EBPP at universities significantly predicted the positive attitude toward EBPP (Nguyen-Thi et al., 2023). This finding requires further investigation, including the expertise of educational curricula at universities in Ukraine, i.e., whether they cover EBPP topics.

Finally, regarding the revealed moderate correlation between the studied outcomes and the difference in their prediction models, we suppose it reflects the previously found non-equivalence of knowing (as a cognitive aspect) and utilizing (as a behavioral aspect) the EBPP approach (Jiménez-Pérez & Vargas-Contreras, 2020). For educational purposes, it supports the idea of seeking an optimal balance between didactic training focused on the transition of knowledge of EBPP and competence training focused on skills development to implement EBPP elements (McHugh & Barlow, 2010).

### Limitations

This study has several limitations associated with sample size and specifics. First, the sample size is rather small, which reflects a widespread problem in collecting data in wartime Ukraine (Pavlova et al., 2024), including among psychologists (Velykodna et al., 2023). Second, gender distribution reflects both the current difficulty of involving men in data collection due to wartime (Lushchak et al., 2024) and gender inequity in mental health positions (Velykodna et al., 2023). One variable (psychologists’ *Belief that they implement EBPP*) used a 5-point Likert scale, measuring by mistake, while other variables used a 7-point Likert scale, which complicated the comparison of β-values obtained in this study.

### Conclusion

Overall, the present study showed that EBPP implementation in Ukraine is only partly associated with predictors extracted from a literature review from previous research, such as EBT utilization, personal therapy and supervision, positive attitudes regarding EBPP, membership in psychological associations, and perceived knowledge. Moreover, psychologists’ belief that they implement EBPP only moderately correlated with its utilization in practice, which highlights the gap between cognitive and behavioral aspects of EBPP utilization. While the perceived quality of university education did not predict the dependent variables, instead of the membership in professional associations, we believe that Ukraine-based mental health professionals would benefit from disseminating EBPP knowledge both through university education and within associations. However, further qualitative research involving Ukrainian psychologists is needed to address the gaps revealed in this study.

## Supplementary Materials

**Table:** 

Type of supplementary materials	Availability/Access
Data	
Data 3-in-1 dataset.	Velykodna and Deputatov (2024)
Data sheet.	Velykodna and Deputatov (2024)
Code	
No code was supplied.	—
Material	
Translation into English.	Velykodna and Deputatov (2024)
Study/Analysis preregistration	
The study was not preregistered.	—
Other	
Codebook.	Velykodna and Deputatov (2024)
Jamovi logs (1–5).	Velykodna and Deputatov (2024)

## Data Availability

The data and Jamovi logs are available at the Open Science Framework repository at Velykodna and Deputatov (2024)

## Appendix

**Table A1:** Variables and Relevant Data Types

Variable	Scale	Data type
Age	Continuous	Number
Gender	Nominal	Options: Male / Female
Current residence.	Nominal	Options: In Ukraine / Abroad
Academic degrees in Psychology (multiple choice).	Nominal	Options: Bachelor / Master / Doctoral Degree
Number of graduated universities.	Continuous	Number
Location of graduated universities.	Nominal	Options: In Ukraine / Abroad
Membership in any psychological association.	Nominal	Options: No / Yes, Ukrainian / Yes, foreign / Yes, international
Field of practice.	Nominal	Options: Education / Science / Mental Health / Social Work / Military / Law / Business / Private practice / Other
Work experience.	Continuous	Number
Quality of university education regarding EBPP.	Ordinal	7-point Likert scale from 1 (“Completely disagree”) to 7 (“Completely agree”)
Learned intervention / method / approach (if available).	Nominal	Options: 23 different methods: EMDR, mindfulness, RTM, art therapy + Other (narrative)
Does this intervention/method have proven efficacy?	Nominal	Options: I don’t know / I don’t use any method / No / Yes
Belief that a psychologist implements EBPP (To what extent do you implement the EBPP approach?).	Ordinal	5-point Likert scale from 1 (Not at all) to 5 (To a large extent) + additional option “I do not know” (0)
Did you ever have personal therapy experience?	Ordinal	Options: no / less than 50 hours / more than 50 hours / more than 100 hours / more than 200 hours / more than 300 hours
Did you ever have supervision experience?		
Intensity of implementing EBPP (Please, indicate how often you utilize the following activities).		
• Updating knowledge about the possibilities and limits of using a particular method or intervention.	Ordinal	7-point Likert scale from 1 (“Less than once in five years”) to 7 (“Daily”)
• Updating knowledge about the peculiarities of working with certain issues, requests, and personality types.		
• Analysis of own practical experience (e.g., through supervision and discussions with colleagues).		
• Analysis of the practical experience of colleagues (e.g., through supervision, discussions with colleagues, and clinical ateliers).		
• Taking into account the sociocultural characteristics of a particular client (and appropriate choice or modification of the approach, method, and technique).		
• Taking into account the personal preferences of the client (and the appropriate choice or modification of the approach, method, and technique).		
Perceived knowledge regarding EBPP.	Ordinal	7-point Likert scale from 1 (“Almost nothing”) to 7 (“Very well”)
Attitude toward EBPP (Please assess each item as important or not important in providing psychological practice).		
• Updating knowledge about the possibilities and limits of using a particular method or intervention.	Ordinal	7-point Likert scale from 1 (“Not important at all”) to 7 (“Very important”)
• Updating knowledge about the peculiarities of working with certain issues, requests, and personality types.		
• Analysis of own practical experience (e.g., through supervision and discussions with colleagues).		
• Analysis of the practical experience of colleagues (e.g., through supervision, discussions with colleagues, and clinical ateliers).		
• Taking into account the sociocultural characteristics of a particular client (and appropriate choice or modification of the approach, method, and technique).		
• Taking into account the personal preferences of the client (and the appropriate choice or modification of the approach, method, and technique).		
